# TCL1 transgenic mice as a model for CD49d-high chronic lymphocytic leukemia

**DOI:** 10.1038/s41375-020-0759-3

**Published:** 2020-02-21

**Authors:** Eva Szenes, Andrea Härzschel, Sarah Decker, Erika Tissino, Justine Pischeli, Julia Christine Gutjahr, Sandra Kissel, Sandra Pennisi, Jan Philip Höpner, Alexander Egle, Nadja Zaborsky, Christine Dierks, Marie Follo, Alexandre Chigaev, Antonella Zucchetto, Richard Greil, Valter Gattei, Tanja Nicole Hartmann

**Affiliations:** 1 Department of Internal Medicine III with Hematology, Medical Oncology, Hemostaseology, Infectiology and Rheumatology, Oncologic Center, Salzburg Cancer Research Institute—Laboratory for Immunological and Molecular Cancer Research (SCRI-LIMCR), Paracelsus Medical University, Salzburg, Austria; 2 Department of Internal Medicine I, Medical Center and Faculty of Medicine, University of Freiburg, Freiburg, Germany; 3 Clinical and Experimental Onco-Hematology Unit, Centro di Riferimento Oncologico di Aviano (CRO) IRCCS, Aviano, Italy; 4 Department of Pathology and Cancer Center, University of New Mexico, Albuquerque, NM USA

**Keywords:** Cancer microenvironment, Chronic lymphocytic leukaemia

## To the Editor:

With the advent of small molecule inhibitors, sustained responses in chronic lymphocytic leukemia (CLL) patients have been achieved. Ibrutinib, an inhibitor of Bruton’s tyrosine kinase (BTK), is a particularly potent treatment for CLL, with overall response rates of 82% in treatment-naive and 43% in relapsed-refractory patients [1]. These responses are accompanied by transient lymphocytosis in 50–70% of patients and a concomitant reduction of tumor burden in lymphoid organs [2]. We have recently demonstrated that the high-risk, CD49d-high CLL subgroup [3] displays reduced lymphocytosis and minor lymph node shrinkage upon ibrutinib treatment [4]. CD49d is the alpha subunit of the integrin VLA-4, which plays an essential role in the tissue retention of cells, orchestrating their adhesion to stromal cells, e.g., follicular dendritic cells (fDCs), which in turn express the VLA-4 ligand VCAM-1. To facilitate this strong adhesion, VLA-4 needs to be activated [5]. We have demonstrated that in CLL the extended, high-affinity conformation of VLA-4 can be triggered by B cell receptor (BCR) stimulation [4]. Notably, upon treatment with ibrutinib, CD49d-high CLL cells maintain residual BCR-mediated inside-out activation of VLA-4, thus explaining the reduced clinical response of CD49d-expressing CLL [4].

Eµ-TCL1-transgenic (TCL1-tg) mice represent a widely used mouse model for CLL [6]. By using this model, here we found that: (i) murine leukemic cells express high levels of surface CD49d; (ii) the BCR-VLA-4 axis in TCL1-tg mice, as investigated upon BCR stimulation and/or exposure to BCR inhibitors, resembles that of the human CD49d-high CLL cohort; (iii) the in vivo distribution of TCL1-tg leukemic cells is dependent on VLA-4-mediated interactions with the microenvironment. Our data demonstrate the value of TCL1-tg mice as a model for CD49d-high CLL and corroborate the importance of retained BCR-induced VLA-4 activation in a clinical setting.

**Fig. 1:**
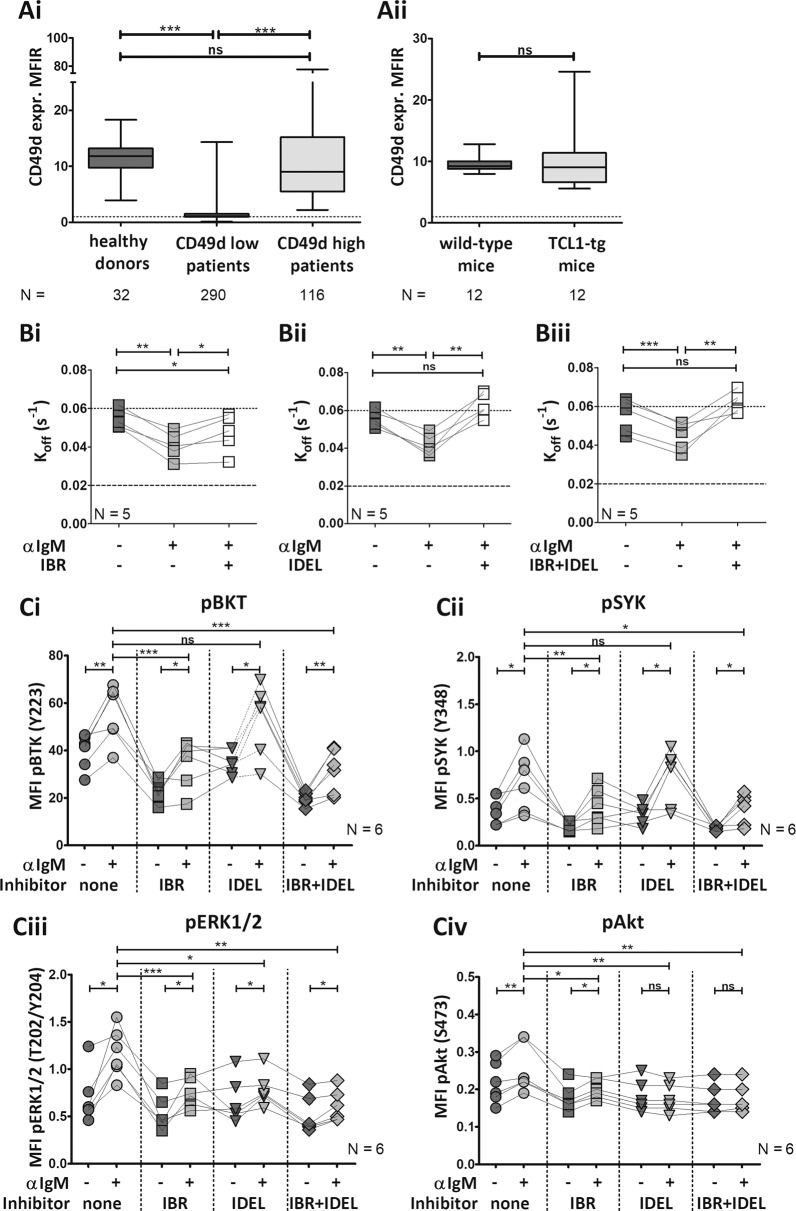
TCL1-tg leukemic cells resemble human CD49d-high CLL cells in their CD49d expression and VLA-4 inside-out signaling. **a** The expression of the rate-limiting alpha subunit of VLA-4, CD49d, was measured by flow cytometry. Samples collected from peripheral blood of age-matched (Ai) healthy donors (*N* = 32), CD49d-low (*N* = 290) or CD49d-high CLL patients (*N* = 116), and (Aii) C57BL/6 J wild-type (*N* = 12) or TCL1-tg mice (*N* = 12) were stained with anti-CD5 and anti-CD19, as well as CD49d-specific or its isotype control antibody. MFIR mean fluorescence intensity ratio (human measurements: arithmetic mean, mouse measurements: geometric mean). Dotted line: MFIR = 1. Unpaired *t* tests were performed by GraphPad Prism5. **b** Purified murine CD5+/CD19+ cells (*N* = 5) were treated for 1 h with/without 1 μM (Bi) ibrutinib (IBR), (Bii) idelalisib (IDEL), or (Biii) both. The dissociation rate (*k*_off_) of VLA-4 and its ligand LDV was determined by real-time flow cytometry. *K*_off_ values are shown upon different stimulation states: resting (negative control), anti-IgM-stimulated (F(ab′)_2_, 10 μg/ml), and pretreated + anti-IgM-stimulated. A *k*_off_ value above 0.06 s^−1^ (dotted line) is considered “low affinity”, while values below 0.02 s^−1^ (dashed line) are “high affinity.” Connecting lines indicate values from the same tumor. Nonlinear fit “Plateau followed by one-phase decay” and paired *t* tests were performed using GraphPad Prism5. **c** Ibrutinib and idelalisib differentially affect BCR signaling in TCL1-tg mice. Splenocytes from TCL1-tg mice (*N* = 6) were pretreated with/without ibrutinib, idealisib or both (1 μM, 1 h). Cells were stained with anti-CD3 for T cell exclusion and with fixable viability dye, followed by a stimulation by anti-IgM (F(ab′)_2_, 10 μg/ml, 5 min). Cells were fixed and permeabilized, stained with phosflow antibodies, and measured by flow cytometry. The mean fluorescence intensity (MFI, geometric mean) values of (Ci) pBTK, (Cii) pSYK, (Ciii) pERK1/2, and (Civ) pAkt of viable CD3-negative cells are shown. To validate whether the CD3-negative cell population corresponds with the leukemic cells, the samples were stained with anti-CD5 and anti-CD19 in parallel. Connecting lines indicate values from the same tumor. Paired *t* tests were performed by GraphPad Prism5. *P < 0.05; **P < 0.01; ***P < 0.001, ns nonsignificant.

First, to compare VLA-4 expression patterns between human CLL cells and TCL1-tg cells, we measured CD49d expression of human or murine healthy B cells and leukemic cells by flow cytometry (antibodies: see Supplementary Table 1). Human blood samples (*N* = 406; Supplementary Table 2) were obtained upon written informed consent and ethical approval. Blood-derived B cells from age-matched healthy individuals (*N* = 32) and CD49d-high CLL cells (*N* = 116) expressed similar levels of CD49d (Fig. 1ai). Previous reports have shown that CD49d-high CLL cells express lower CD49d levels than healthy B cells. However, our healthy donor group consisted of strictly age-matched individuals (average of healthy cohort: 65.8; CLL cohort: 69.63 years), indicating that CD49d expression might be reduced by age in humans. Moreover, CD49d expression of blood-derived B cells from age-matched C57BL/6J wild-type (WT) mice was similar to that of leukemic cells from TCL1-tg mice (Fig. 1aii). To investigate whether the expression of the VLA-4 subunits (CD49d and CD29) had an organ-specific pattern, we measured B cells and leukemic cells of spleen (SPL), bone marrow (BM), lymph nodes (LN), blood (BL), liver (LIV), and the peritoneal cavity (PC) from WT and TCL1-tg mice. Remarkably, expression levels of both integrin subunits were similar in all organs. CD49d mean fluorescence intensity ratio (MFIR) was comparable in WT and TCL1-tg mice (mean MFIR of WT = 9.893; TCL1-tg = 9.710) (Supplementary Fig. 1a), while the expression of the CD49d-coupled beta subunit CD29 (beta1) was higher in TCL1-tg mice (mean MFIR of WT = 7.261; TCL1-tg = 21.920) (Supplementary Fig. 1b). Altogether, our data demonstrate that the TCL1-tg model can be used as a murine surrogate for the CD49d-high CLL cohort, with stable expression. As expression levels in the spleen and blood were comparable, and their cellular compositions are highly similar, we used murine splenocytes in subsequent experiments.

To relate the CD49d expression pattern to function, we investigated the mechanistic basis of VLA-4 activation by BCR stimuli. In human CLL, BCR-induced VLA-4 activation can be detected by the conformation-sensitive anti–human CD29 antibody HUTS-21 [4, 7], which does not recognize the murine epitope. Therefore, here we used the small molecule VLA-4 ligand LDV [8] and measured its binding to leukemic cells by flow cytometry as outlined in Supplementary Fig. 2. With this experimental layout, we obtained real-time information about the kinetics of VLA-4 affinity regulation resulting in enhanced ligand binding. The speed by which unlabeled LDV replaces its FITC-conjugated counterpart (*k*_off_) is used to determine integrin affinity [8]. If VLA-4 and its ligand bind with high affinity, dissociation of LDV-FITC is slower and the replacement time is longer, resulting in lower *k*_off_ values (below 0.02 s^−1^). Conversely, low VLA-4-ligand affinity leads to fast LDV-FITC dissociation, shorter replacement time, and higher *k*_off_ values (above 0.06 s^−1^). When purified TCL1-tg leukemic cells were stimulated with anti-mouse-IgM, VLA-4 affinity increased, with a decrease in *k*_off_ from 0.055 to 0.04 s^−1^. This shows for the first time that in TCL1-tg mice BCR stimulation induces changes in VLA-4 conformation. Ibrutinib pretreatment did not fully prevent this inside-out activation of VLA-4 (Fig. 1bi), recapitulating the observations in human CD49d-high CLL cells [4]. Next, we treated the cells with the phosphoinositide-3-kinase delta (PI3Kδ) inhibitor idelalisib [9]. Treatment of leukemic cells with idelalisib (Fig. 1bii) and the ibrutinib/idelalisib combination (Fig. 1biii) was highly effective with no difference between the affinities of unstimulated compared with stimulated + idelalisib-treated or stimulated + combination-treated cells. In human CLL, neither ibrutinib nor idelalisib is sufficient on its own to prevent inside-out signaling via the BCR [4], suggesting that the stimulation can bypass both BTK and PI3Kδ. In TCL1-tg mice, PI3Kδ seems to be a dominant molecule that mediates inside-out signaling between the BCR and VLA-4.

To test that the residual BCR-induced VLA-4 activation was not a result of insufficient inhibition by ibrutinib, we measured the phosphorylation of signaling molecules of the BCR pathway in TCL1-tg splenocytes treated with/without BCR inhibitors by phosflow. Ibrutinib and idelalisib showed specific and expected patterns of inhibition. Single-agent ibrutinib treatment significantly reduced the phosphorylation of BTK (Fig. 1ci) and spleen tyrosine kinase (SYK, Fig. 1cii) after BCR stimulation. Extracellular signal-regulated kinase 1/2 (ERK1/2, Fig. 1ciii) phospho signal was decreased by both inhibitors. The phosphorylation of Akt (Fig. 1civ), the downstream signaling component of PI3K, was efficiently inhibited by idelalisib. Both inhibitors showed expected inhibition, therefore, the residual BCR-mediated VLA-4 activation in ibrutinib-treated cells was not a result of suboptimal BTK blocking.

**Fig. 2:**
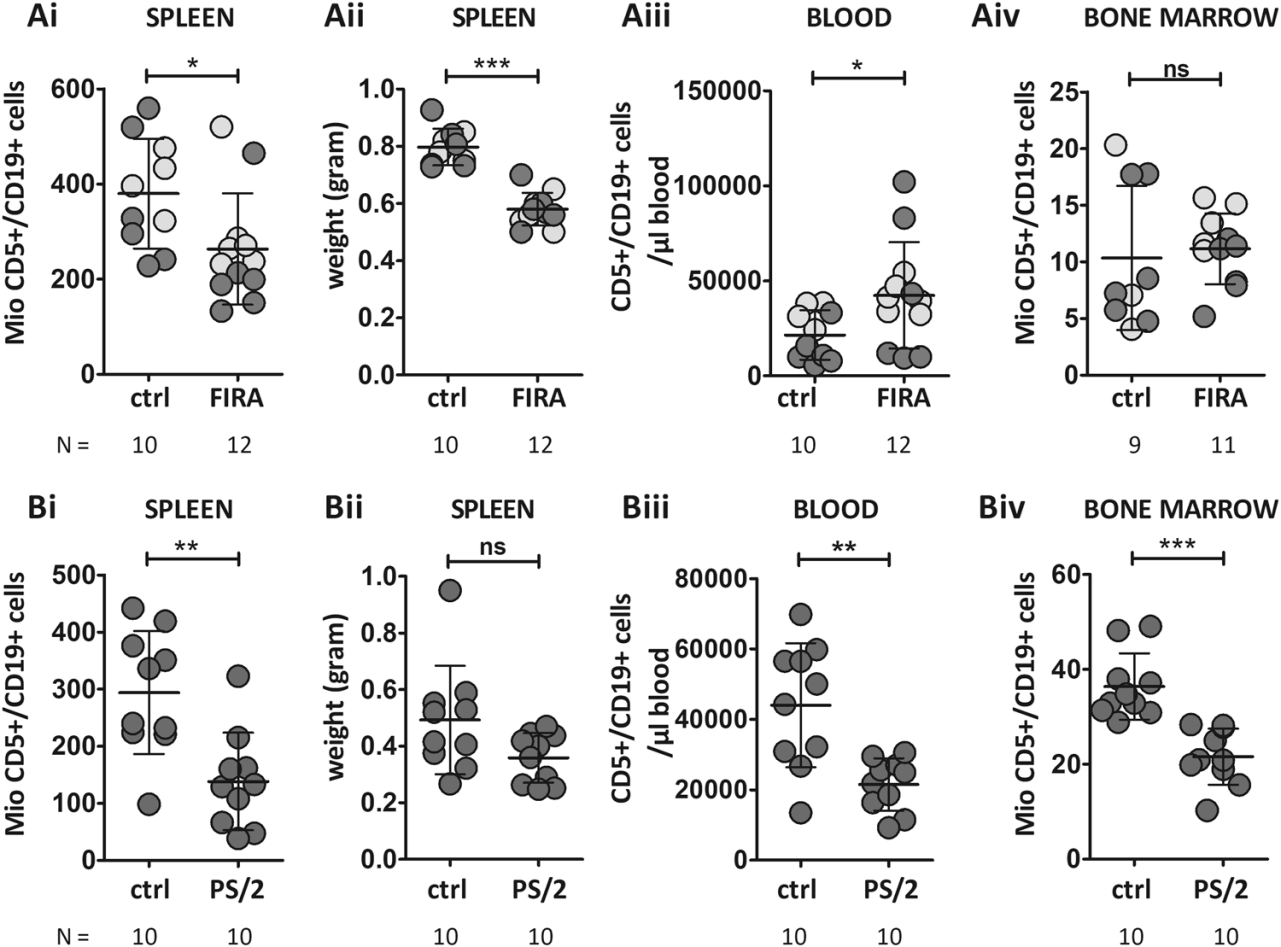
Inhibition of CD49d in mice transplanted with TCL1-tg splenocytes prevents the engraftment of leukemic cells short-term in the spleen and long-term in the spleen and bone marrow. **a** Wild-type C57BL/6J mice were transplanted intravenously with ~5 Mio splenocytes from a TCL1-tg mouse, then treated with vehicle (0.9% NaCl, 1% HP-β-CD) (*N* = 10, bone marrow *N* = 9) or the small molecule inhibitor firategrast (*N* = 12), 30 mg/kg/day in drinking water, starting two (light gray) or seven (dark gray) days post transplantation. Tumor development was monitored by measuring CD5 and CD19 from blood samples from the tail vein twice a week. Mice were sacrificed 21 days after transplantation. Organ infiltration and tumor burden were measured by cell counting (EVE automatic counter) and flow cytometry, with CD5/CD19 staining identifying leukemic cells. **b** Wild-type C57BL/6J mice were transplanted intraperitoneally with 25 Mio CLL cells from a TCL1-tg mouse, then treated with the CD49d-blocking antibody PS/2 (*N* = 10), 10 mg/kg, or isotype control (*N* = 10), injected intraperitoneally twice a week, starting 7 days post transplantation. Mice were sacrificed 5 weeks after treatment start (6 weeks post transplantation). Tumor burden was determined by CD5/CD19 staining and manual cell counting. The absolute numbers of malignant cells from the (Ai, Bi) whole spleen, along with its (Aii, Bii) weight, from (Aiii, Biii) blood (normalized to μl) and from the (Aiv, Biv) bone marrow (extrapolated from the femora to the whole bone marrow as described in Supplementary Methods) were determined. Unpaired *t* tests were performed by GraphPad Prism5. Error bars represent standard deviation (SD). *P < 0.05; **P < 0.01; ***P < 0.001, ns nonsignificant.

Having examined the connection between the BCR and VLA-4 in vitro, we next investigated the importance of VLA-4 in engraftment and early disease onset of TCL1-tg-driven murine leukemia in vivo. Therefore, we transplanted WT mice with primary TCL1-tg splenocytes [10] and treated the recipients with vehicle or the small molecule CD49d-inhibitor firategrast [11]. Recipient mice were sacrificed and their organs analyzed by flow cytometry 21 days post transplantation. Firategrast-treated mice showed an overall reduction of leukemic cells in the spleen (Fig. 2ai), accompanied by significant spleen weight reduction (Fig. 2aii). Since leukemic cells need to enter the follicles in the white pulp of the spleen and access fDCs expressing VCAM-1 and providing proliferative signals [12], VLA-4 inhibition prevents the cells from staying in this supportive niche. Indeed, we found a significant increase of CD5+/CD19+ cells in the blood (Fig. 2aiii) upon VLA-4 inhibition, without changes in tumor infiltration in the BM (Fig. 2aiv). This finding was surprising given the prominent role of VLA-4 in CLL retention in the BM [13], but at this stage of engraftment the amount of leukemic cells was still low, and TCL1-tg tumor growth is driven by the spleen, especially in early stages [14].

To investigate a more advanced disease stage, we repeated the treatment study using the inhibitory rat-anti-mouse CD49d antibody PS/2 (Fig. 2b, treatment schedule: see legend). For long-term treatment, we switched to antibody therapy because firategrast has a fast turnover in the organism, although the in vitro inhibitory effects on VLA-4 ligand binding of the two substances were comparable (confirmed by adhesion assays, data not shown). The mice were sacrificed 6 weeks post transplantation. Analysis of the organs revealed decreased tumor burden in the spleen (Fig. 2bi), along with slightly decreased spleen weight (Fig. 2bii). At this stage, a significant decrease of the tumor load in the blood of PS/2-treated mice was also observed (Fig. 2biii); furthermore, tumor burden in the BM of PS/2-treated mice was significantly decreased (Fig. 2biv). Further investigations are needed to determine whether the redistribution by CD49d inhibition might overcome drug resistance or improve the effectiveness of other therapeutic agents by detaching CLL cells from VCAM-1-expressing stromal cells.

Based on our findings, we propose that TCL1-tg mice represent an adequate model for CD49d-high CLL patients, and provide evidence that BCR-induced VLA-4 inside-out activation remains functional upon treatment with ibrutinib, being in turn hampered upon exposure to idelalisib. This indicates that PI3K, rather than BTK, is an essential part of the signaling between the BCR and VLA-4 in the TCL1-tg model, in part confirming the observation made in humans [4]. Furthermore, we specified the role of CD49d in disease development by directly targeting this integrin in vivo, which led to a redistribution of TCL1-tg cells transplanted into WT mice. From a clinical perspective, we suggest that VLA-4 inhibition or additional targeting of BCR pathway molecules, e.g., by PI3K inhibitors other than idelalisib to avoid its severe side effects [15] and potentially targeting different isoforms, can be promising additions to ibrutinib therapy for high-risk CD49d-high CLL patients. This could help to overcome residual BCR-mediated signaling bypassing BTK and mediating tissue retention facilitated by VLA-4.

## Supplementary information

Supplemental material
