# Supplementary material for: TCL1 transgenic mice as a model for CD49d-high chronic lymphocytic leukemia
Source: Leukemia. 2020 Feb 21;34(9):2498–502. doi: 10.1038/s41375-020-0759-3 (PMC7449868; doi:10.1038/s41375-020-0759-3)
Supplement: Supplementary file 1 — Supplemental material [file 41375_2020_759_MOESM1_ESM.pdf]

Supplemental Methods

Mice

All mice used in this study were on C57BL/6J background. TCL1-tg mice of both sexes were used, the age of the primary TCL1-tg mice when sacrificed due to disease progression varied between 215 and 467 days. For the transplantation experiments shown in Figure 2, female C57BL/6J mice between 8 and 12 weeks of age were used as recipients.

No randomization was performed before start of the firategrast treatment (Figure 2A), as all animals were injected with the same amount of tumor cells, and could thus be considered equal at treatment start, and randomization not obligatory.

For the PS/2 treatment experiment (Figure 2B), CLL counts in the blood were measured before treatments start, and the mice were distributed equally among the two groups with regard to the blood tumor load. There were only minor variations in tumor load between the mice at that timepoint.

Total cell numbers were extrapolated from the femora to the whole bone marrow by multiplying with 10.6 as described in (1).

Statistics

Sample sizes were estimated using the general standards for power and significance (Power (1-beta): 0.8, error rate (alpha): 0.05). Means and standard deviations were estimated on basis of similar previously performed experiments or published literature.

All statistical data analysis was performed using GraphPad Prism5. Figure 1A and Supplemental Figure 1 show box and whiskers plots, with the box indicating the 25th to 75th percentiles, the median indicated as line in the middle of the box and whiskers from min to max. All other graphs display individual values. Normal distribution was tested for all data sets, and two-tailed statistical analysis performed as individually described in the figure legends.

Supplemental Figure 1

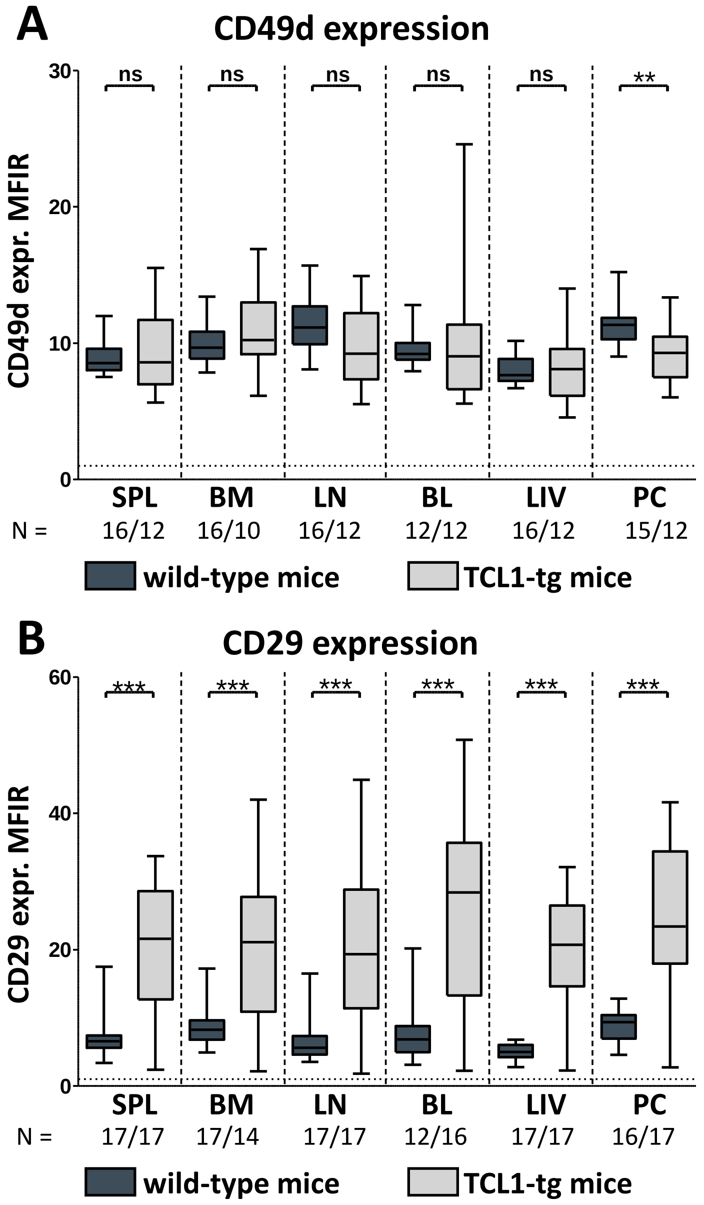

Suppl. Figure 1. ***CD49d and CD29 expression of CD5+/CD19+ cells from TCL1-tg mice compared to B cells from wild-type mice.*** The expression of the alpha and beta subunits of the integrin VLA-4, (**A**) CD49d and (**B**) CD29 was measured by flow cytometry. Wild-type or TCL1-tg mice (N=10–17) were sacrificed and cells from the following compartments were collected: spleen (SPL), bone marrow from the femora (BM), four peripheral lymph nodes (LN), heart blood (BL), liver (LIV), and peritoneal cavity (PC). Samples were stained with anti-CD5 and anti-CD19 antibodies, as well as anti-CD49d and anti-CD29 or their corresponding isotype control antibodies. MFIR: mean fluorescence intensity ratio (geometric mean). Dotted line: MFIR=1. Unpaired t-tests were performed using GraphPad Prism5. *=P<0.05, **=P<0.01, ***=P<0.001, ns=non-significant.

Supplemental Figure 2

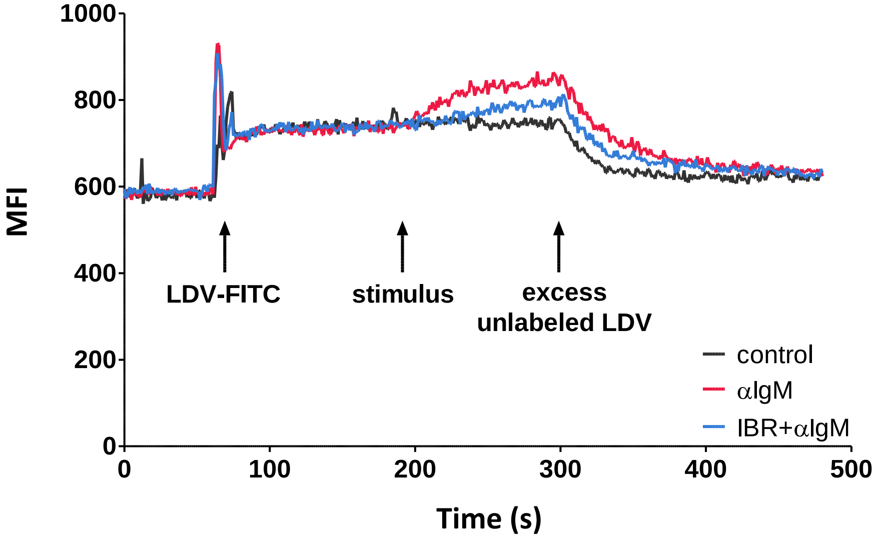

Suppl. Figure 2. ***Flow cytometric measurement of VLA-4 inside-out signaling in CD5+/CD19+ cells from TCL1-tg mice.*** Kinetics of binding and dissociation of the peptide derived from the LDV sequence of the VLA-4-binding region of fibronectin (LDV). Representative measurement of CD5+/CD19+ splenocytes of a TCL1-tg mouse is plotted as median fluorescence intensity (MFI) versus time after sequential additions of LDV-FITC, cell stimulation and unlabeled LDV-block. Three types of stimuli are depicted: PBS as negative control (grey), anti-IgM (F(ab')2, 10 µg/ml; red) and ibrutinib+anti-IgM (IBR: 1 µM, 1-hour pre-treatment; blue). The dissociation rate (k_{off}, s^{-1}, representing the affinity of the receptor and its ligand) of VLA-4 and LDV can be calculated as the K value of the one-phase decay curve after the unlabeled LDV has been added in excess.

Supplemental Table 1

List of antibodies

Target antigen / Name of reagent	Fluorescent dye	Target species	Company	Cat#	Experiment
CD5	FITC	hu	Beckman Coulter (Brea, CA, USA)	A08932	FC
CD19	PE/Cy7	hu	Beckman Coulter (Brea, CA, USA)	IM3628	FC
CD49d	PE	hu	BD Pharmingen (San Jose, CA, USA)	555503	FC
isotype control (mouse IgG1 κ)	PE	---	BD Pharmingen (San Jose, CA, USA)	555749	FC
CD5	APC/eFluor 780	mo	Affymetrix eBioscience (Waltham, MA, USA)	47-0051-82	FC
CD19	eFluor 450	mo	Affymetrix eBioscience (Waltham, MA, USA)	48-0193-82	FC
CD49d	FITC	mo	BioLegend (San Diego, CA, USA)	103606	FC
isotype control (rat IgG2b κ)	FITC	---	BioLegend (San Diego, CA, USA)	400606	FC
CD29	PE/Cy7	mo	BioLegend (San Diego, CA, USA)	102222	FC
isotype control (Armenian hamster IgG)	PE/Cy7	---	BioLegend (San Diego, CA, USA)	400922	FC
Beta7	PerCP/Cy5.5	mo	BioLegend (San Diego, CA, USA)	321220	FC
isotype control (rat IgG2a κ)	PerCP/Cy5.5	---	BioLegend (San Diego, CA, USA)	400532	FC
CD5	PE/Cy5	mo	BioLegend (San Diego, CA, USA)	100610	TS
CD19	PE	mo	BioLegend (San Diego, CA, USA)	115508	TS
anti-VLA-4 clone PS/2	unlabeled	hu, mo	Bio X Cell (West Lebanon, NH, USA)	BE0071	TS
Rat IgG2b isotype clone LTF-2	unlabeled	---	Bio X Cell (West Lebanon, NH, USA)	BE0090	TS
Btk (pY223)/Itk (pY180)	PE	hu, mo	BD Biosciences (San Jose, CA, USA)	562753	PF
ERK1/2 (pT202/pY204)	AF488	hu, mo	BD Biosciences (San Jose, CA, USA)	612592	PF
Syk (pY348)	AF488	hu, mo	BD Biosciences (San Jose, CA, USA)	560081	PF
Akt (pS473)	AF647	hu, mo	BD Biosciences (San Jose, CA, USA)	560343	PF
CD3	BV421	mo	BD Biosciences (San Jose, CA, USA)	740014	PF
fixable viability dye	FVS700	mo	BD Biosciences (San Jose, CA, USA)	564997	PF
CD5	FITC	mo	BD Biosciences (San Jose, CA, USA)	553020	PF
CD19	APC	mo	BD Biosciences (San Jose, CA, USA)	550992	PF

FC: flow cytometry
TS: treatment study
PF: phosflow

Supplemental Table 2

Patient characteristics

Ref	Patient data (total number = 406)		Healthy donors (total number = 32)	
	Age (years)	69.63 (range 39–88)	Age (years)	65.8 (range 60–71)
	Male gender (%)	57.64	Male gender (%)	43.75
	**Risk factors**			
	IgHV unmutated (%)	29.06		
(2)	ZAP-70 high expressing (%)	36.45		
(3)	CD49d high expressing (%)	28.57		
(4)	CD38 high expressing (%)	21.92		
(5)	FISH high-risk, either del17p or del11q (%)	11.58		

Supplemental References

1. Boggs DR. The total marrow mass of the mouse: a simplified method of measurement. Am J Hematol. 1984;16(3):277-86.

2. Letestu R, Rawstron A, Ghia P, Villamor N, Boeckx N, Boettcher S, et al. Evaluation of ZAP-70 expression by flow cytometry in chronic lymphocytic leukemia: A multicentric international harmonization process. Cytometry B Clin Cytom. 2006;70(4):309-14.

3. Bulian P, Shanafelt TD, Fegan C, Zucchetto A, Cro L, Nuckel H, et al. CD49d is the strongest flow cytometry-based predictor of overall survival in chronic lymphocytic leukemia. J Clin Oncol. 2014;32(9):897-904.

4. Brachtl G, Sahakyan K, Denk U, Girbl T, Alinger B, Hofbauer SW, et al. Differential bone marrow homing capacity of VLA-4 and CD38 high expressing chronic lymphocytic leukemia cells. PLoS One. 2011;6(8):e23758.

5. Dohner H, Stilgenbauer S, Benner A, Leupolt E, Krober A, Bullinger L, et al. Genomic aberrations and survival in chronic lymphocytic leukemia. N Engl J Med. 2000;343(26):1910-6.
